# Atherosclerotic cardiovascular disease landscape in Singapore

**DOI:** 10.3389/fcvm.2024.1342698

**Published:** 2024-04-24

**Authors:** Ching-Hui Sia, Oliver Simon, Poay-Huan Loh, Kian Keong Poh

**Affiliations:** ^1^ Department of Cardiology, National University Heart Centre Singapore, Singapore, Singapore; ^2^ Yong Loo Lin School of Medicine, National University of Singapore, Singapore, Singapore; ^3^ Medical Affairs, Novartis (Singapore) Ltd Pte, Singapore, Singapore; ^4^ Division of Cardiology, Department of Medicine, Ng Teng Fong General Hospital, Singapore, Singapore

**Keywords:** cardiovascular disease, atherosclerosis, disability-adjusted life year, global disease burden, medication adherence, Singapore

## Abstract

Cardiovascular disease (CVD) is the leading cause of death worldwide, accounting for over one-third of all deaths in Singapore. An analysis of age-standardized mortality rates (ASMR) for CVD in Singapore revealed a deceleration in the initial rapid decline in ASMR. A decrease in smoking prevalence may have contributed to the initial rapid decline in ASMR. Furthermore, other major risk factors, such as diabetes mellitus, hypertension, elevated low-density lipoprotein levels, and obesity, are steadily rising. Singapore's CVD economic burden is estimated to be 8.1 billion USD (11.5 billion SGD). The burden of CVD can only be reduced using individual and population-based approaches. Prevention programs must also be developed based on an understanding of risk trends. Therefore, this article attempts to capture the burden of CVD, trends in risk factor control, preventive care, disparities, and current unmet needs, particularly in atherosclerotic cardiovascular disease management in Singapore.

## 1 Introduction

Cardiovascular diseases (CVD) have the unfortunate distinction of being the leading cause of death worldwide. Additionally, they contribute to rising healthcare costs and disabilities. Despite the use of effective preventive strategies and advances in care, global trends indicate that CVD cases have nearly doubled from 271 million in 1990 to 523 million in 2019, with a steady increase in deaths caused by CVD from 12.1 million in 1990 to 18.6 million in 2019 (1).

Over half of the worldwide CVD-related deaths occurred in Asia in 2019. CVD was responsible for 35% of the deaths in Asia in 2019. From 1990 to 2019, the mortality has increased by 5.2 million. CVDs are increasing due to various risk factors, including unhealthy diets, obesity, smoking, diabetes, dyslipidemia, hypertension, and the ageing population. The most common cause of death in the regions of southern, central, and western Asia is ischemic heart disease (IHD), whereas in southern, eastern, and central Asia, stroke is the most common cause of death (2). According to recent data published in 2021, one-third of deaths in Singapore are caused by cardiovascular disease, with myocardial infarction (MI) and stroke being the leading causes of death (3, 4).

Several studies have shown that ethnicity has a significant impact on CVD severity. The Singaporean population consists mainly of three major ethnic groups: Chinese (74.3%), Malays (13.4%), and Indians (9.0%). There are differences in the prevalence of risk factors among these groups as well as a disproportionate burden of diseases among them (5). It has been reported that the incidence of MI is greater among Indians and Malays as opposed to Chinese individuals. The risk factors include differences in environmental risk factors (diet, smoking, and physical activity) arising from cultural variations, differences in abdominal obesity, insulin resistance, and thrombogenic factors (6). Singapore's cardiovascular landscape is unique because of its ethnic diversity, socioeconomic environment, aging population, and developmental transition.

Singapore has experienced a rapid decline in age-standardized mortality rates (ASMR) of cardiovascular disease due to preventive public health initiatives undertaken decades ago. However, the decline in ASMR has slowed since the year 2000. A deeper analysis of the changing trends of various risk factors is necessary to achieve further decline in rates (7). Understanding the burden and epidemiological characteristics of CVD in Singapore will enable the development, adaptation, and implementation of effective policies and strategies. There is a lack of literature on CVD trends in Singapore, particularly atherosclerotic cardiovascular disease (ASCVD). In this article, we examine the CVD landscape in Singapore, with a focus on ASCVD. We review the burden of CVD, trends in risk factor control, preventive care, disparities, and current unmet needs in the management of ASCVD in Singapore. This review particularly focusses on elevated low-density lipoprotein cholesterol (LDL-C), which is unanimously recognized as a major modifiable risk factor in the management of ASCVD (8).

## 2 Methodology

An electronic search was performed in the PubMed and Embase databases using relevant keywords related to the burden, risk factors, and management of ASCVD in Singapore. Evidence was gathered from systematic reviews, randomized controlled trials, consensus papers, and expert opinions published in the last five years on the cardiovascular landscape in Singapore. The findings of the literature search were shared among the authors who reviewed the material and included relevant information in this review.

## 3 Burden of disability and death—key indicators

**Figure 1:**
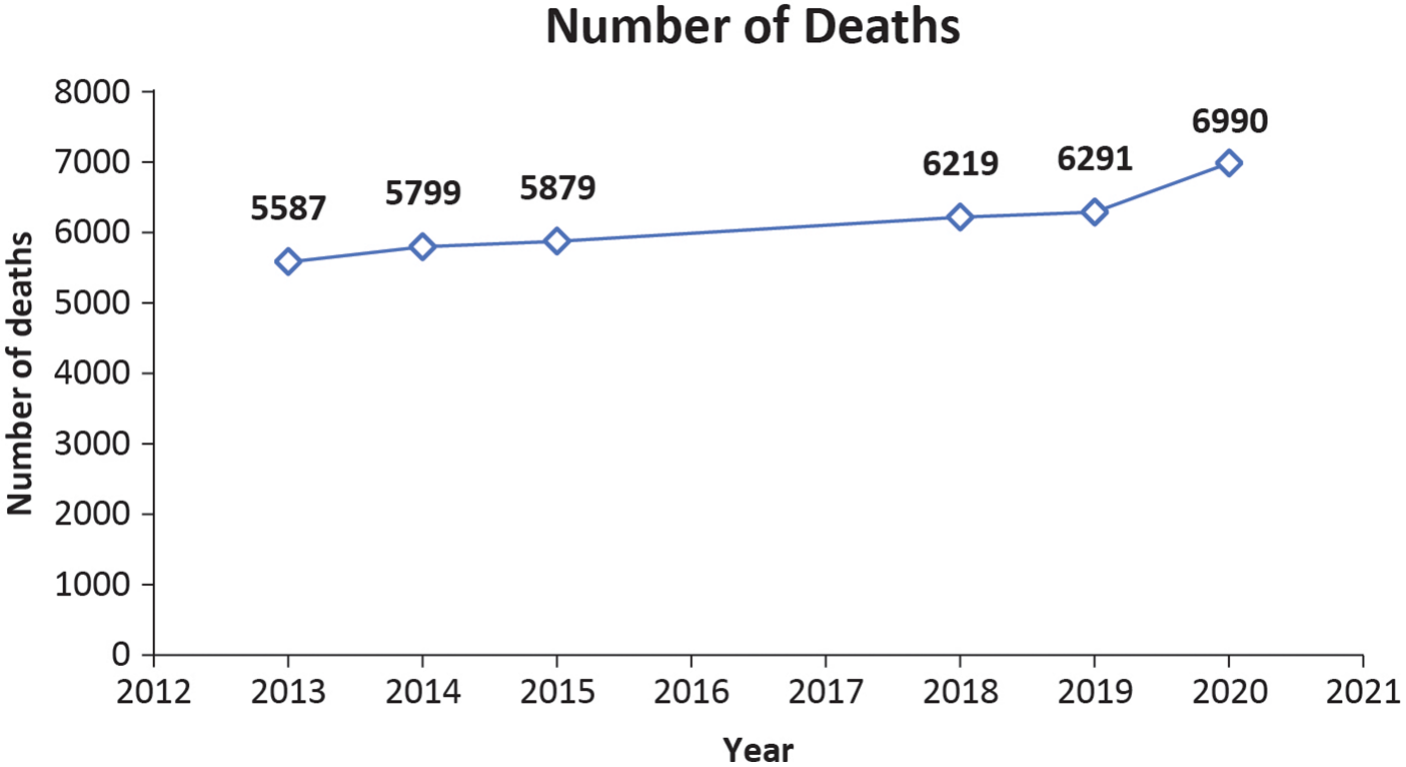
The line graph shows the number of deaths due to CVD from 2013 to 2020 (4, 9).

As shown in Figure 1, the number of CVD-related deaths in Singapore has consistently increased from 2013 to 2020 (4, 9). The proportion of premature deaths (defined as the death of a person aged <70 years) resulting from CVD is higher (nearly 39%) in Asia in general than in the United States (23%), Europe (22%), and globally (34%) (2).

**Figure 2:**
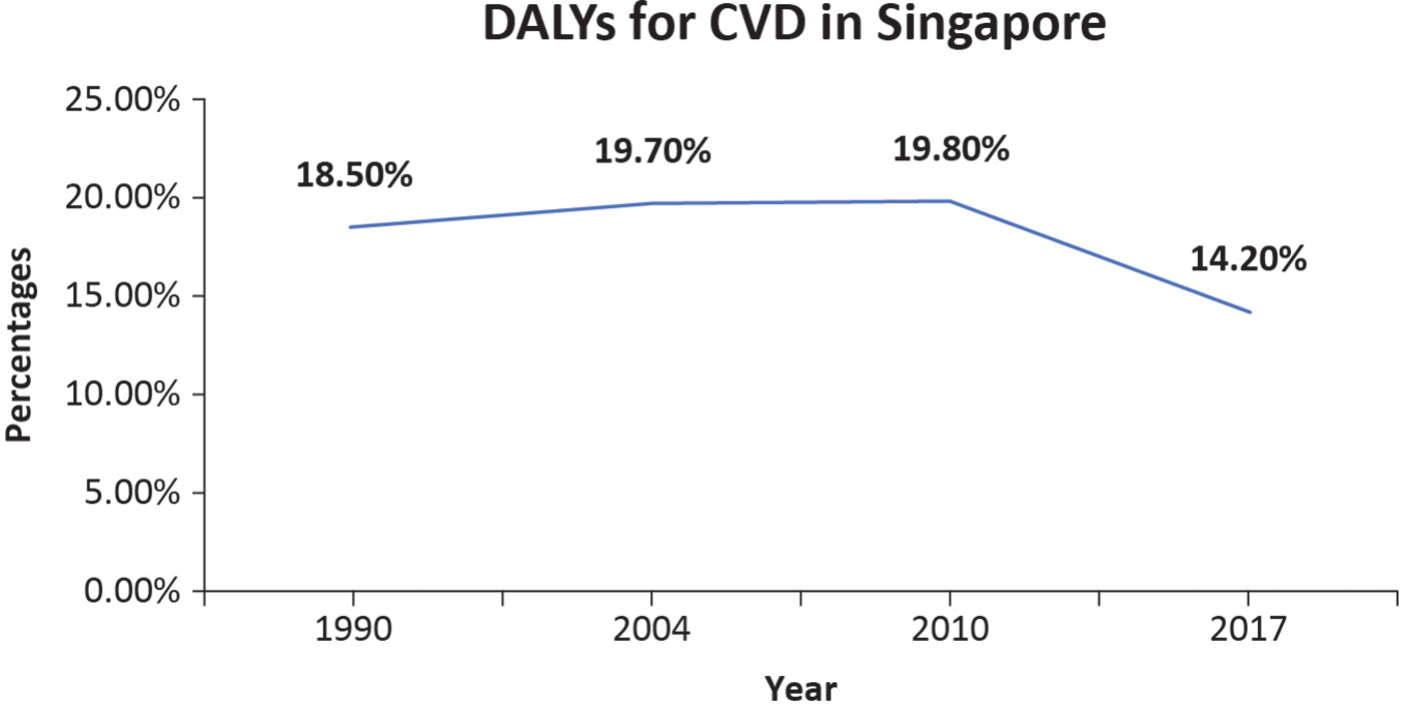
The line graph shows the percentage of CVD-related DALYs among total DALYs in Singapore (3, 11, 12).

CVD can also lead to significant disability, measured as disability-adjusted life years (DALYs) (10). DALYs related to CVD in Singapore are shown in Figure 2. The total percentage change in CVD-related DALYs in Singapore has changed from 18.50% to 14.20% between 1990 and 2017 (3, 11, 12). DALYs have declined over time, indicating some success in preventing and treating CVD.

An additional indicator of CVD burden is ASMR. Between the period 2000 and 2010, ASMR decreased by −4.2% in males and −5.2% in females. ASMR decreased between 2010 and 2016; however, the annual percentage change was −3.4% for males and −5.0% for females. Furthermore, the latest annual change in percentage was −2.4% for males and −5.8% for females. Hence, from 2000 onwards, ASMR continued to decrease with a greater annual percentage reduction initially, followed by a relatively lesser reduction after 2010 (7).

## 4 Atherosclerotic landscape: recent trends of risk factors

ASCVD is a chronic condition with a protracted asymptomatic period spanning several decades and is associated with significant morbidity and mortality. It encompasses coronary heart disease (CHD), cerebrovascular disease (stroke), and peripheral arterial disease, all of which have atherosclerotic origins (13). ASCVD is the most common type of CVD in Asia and its prevalence is increasing in many countries (2). Many factors contribute to this pattern, including a rapidly aging population, increasingly unhealthy lifestyle habits, clinical risk factors, psychosocial factors, and public health transitions (14, 15). Over the past few decades, Singapore has experienced a similar rapid increase in CHD rates (14).

Increasing age, male sex, and family history of premature CHD are non-modifiable risk factors for CHD. Modifiable risk factors include cigarette smoking, high LDL-C, low high-density lipoprotein cholesterol, hypertension, chronic kidney disease, sedentary lifestyle, obesity, stress, excessive alcohol consumption, and diabetes (16). Analyzing the trends among the major risk factors can reveal areas that have improved and those that require focused efforts to reduce the overall burden of ASCVD.

### 4.1 Age and sex

Both, age and sex are the risk factors for CVD (15). Owing to decreasing fertility rates and increasing life expectancy (LE), Singapore is facing the challenge of an aging population (14, 17). Despite the higher incidence of significant CHD in men, it increases in women after menopause, with the risk equalizing for both sexes in the seventh decade (16, 18).

### 4.2 Diet and lifestyle

Increasing affluence has led to significant changes in food choices and practices among the Singaporean population. Consumption of processed foods and high-sugar foods, as well as a decrease in vegetable and fruit intake, are some of the commonly observed deleterious dietary habits (14). Data from the National Nutrition Surveys conducted in 1998, 2004, and 2010 reveal an excessive intake of calories by adult Singaporeans, with an increasing percentage of total fat, saturated fat, and cholesterol being part of their diet (14, 19, 20). The recommended caloric intake for their age and gender was met or exceeded by approximately 50% of the population (14).

Despite an increasing trend of excessive food intake, particularly foods rich in fats and sugars, many people do not exercise (21). Exercise improves myocardial oxygen uptake and coronary artery diameter, and reduces the risk of hypertension, diabetes, and hypercholesterolemia (16). The age-standardized rates of regular leisure-time exercise among Singaporeans decreased from 80.9% in 2017 to 76.8% in 2020. Many people reported that commuting to work was their only physical activity (21).

### 4.3 Tobacco use

**Figure 3:**
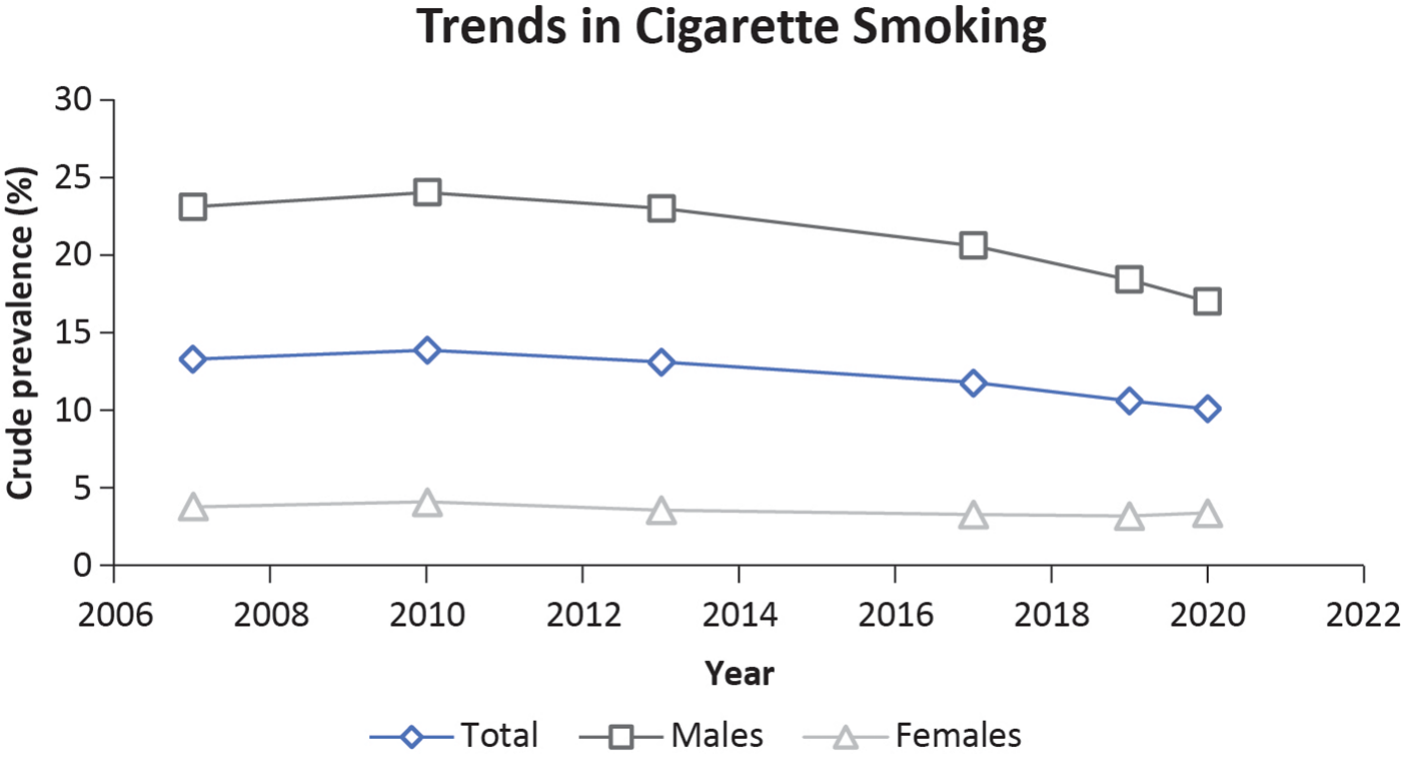
Trends in cigarette smoking among Singaporeans aged 18–74 years (21).

In Singapore, the proportion of adults aged 18–74 years who smoked cigarettes have decreased over time. Men are more likely to smoke than are women (21). Smoking is a well-established risk factor for incident stroke and acute AMI. Yeo *et al*. reported that AMI and stroke patients have a much higher prevalence of smoking, i.e., 43.1% and 38.6% respectively when compared with the national smoking prevalence in Singapore (24%) (22). Figure 3 shows the smoking trends among men and women in Singapore. Among men, a trend of steady decline in smoking was observed. Considering the declining prevalence of smoking in Singapore, other aspects such as lifestyle, genetics, and their interactions need to be explored further (23).

### 4.4 Obesity

**Figure 4:**
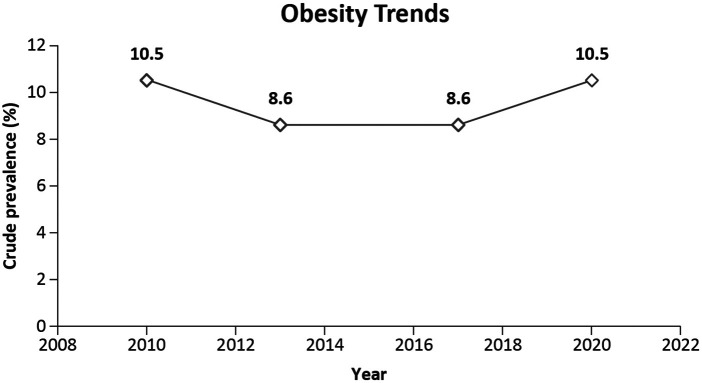
Trends in the prevalence of obesity among Singaporeans aged 18–74 years (21).

Abnormalities in lipid metabolism, inflammation, endothelial dysfunction, adipokine imbalance, inflammasome activation, and insulin resistance are the risk factors that underlie the relationship between obesity and atherosclerosis. Individuals of Asian and South Asian ethnicities tolerate the accumulation of abdominal visceral adipose tissue poorly, manifesting as glucose intolerance at the lower abdominal circumference, and are thus at an elevated risk of developing cardiovascular disease. Approximately 12% of adults aged 30–59 years in Singapore were obese between 2019 and 2020 (24). Approximately 13% of adults have a high-risk body mass index (BMI) (BMI ≥ 27.5 kg/m^2^). The prevalence of abdominal obesity increased with age, with the highest prevalence among adults aged 60–74 years (56.9%). Compared with Chinese (16.1%), Malay (38.7%) and Indian individuals (31.8%) had higher proportions of high-risk BMIs. Education level also seemed to be inversely related to the incidence of high-risk BMI (21). Figure 4 indicates the trends in the prevalence of obesity in Singapore.

### 4.5 Dyslipidemia

**Figure 5:**
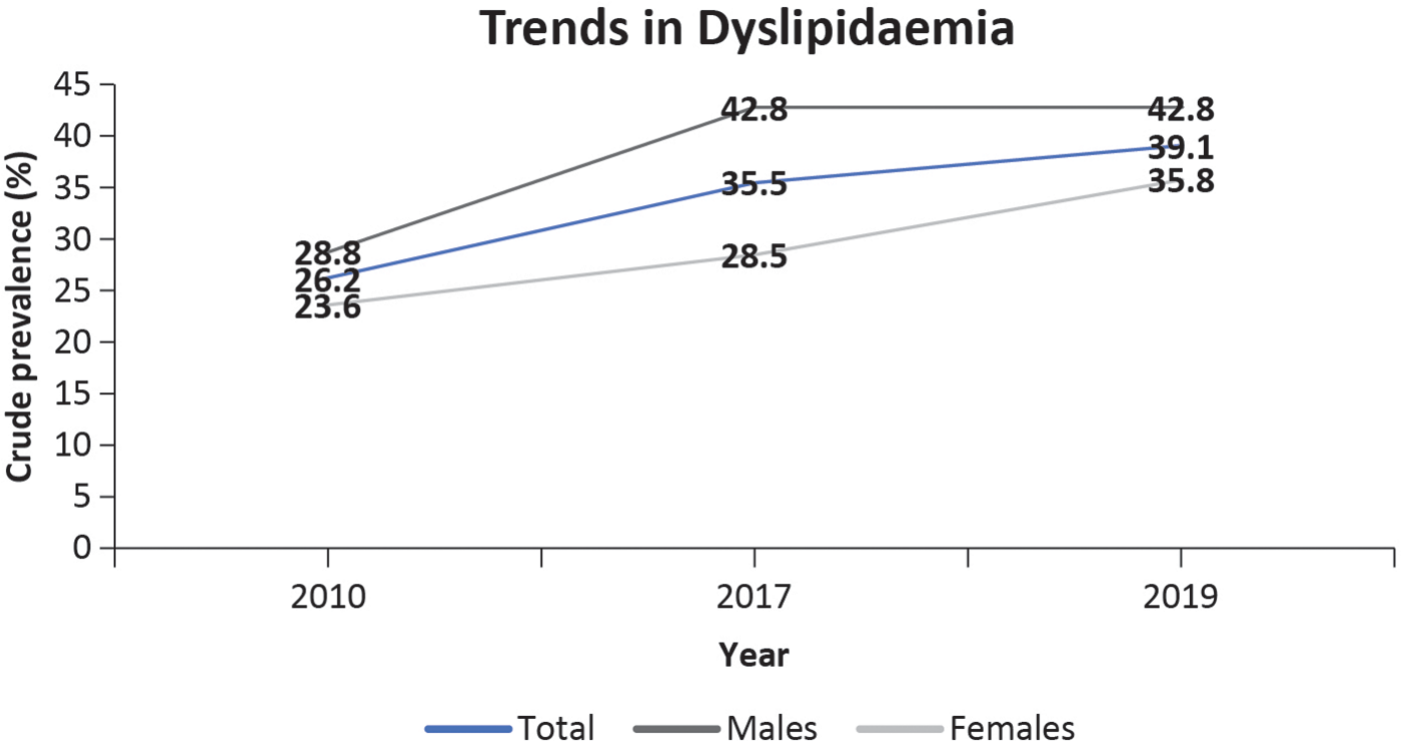
Trends in dyslipidemia in Singaporeans aged 18–74 years (21).

Dyslipidemia is a risk factor for coronary artery disease. There is a well-established relationship between atherosclerotic-ischemic CVD and total cholesterol levels (25). According to age-standardized data, the prevalence of dyslipidemia increased from 33.8% in 2017 to 36.9% in 2019–2020 (21). There is an estimated prevalence of 48.1% in adult Singaporeans with dyslipidemia, 20% of whom are unaware that they have the disease, and 40% of whom would qualify for lipid-lowering therapy (21). Figure 5 shows an increasing trend in dyslipidemia in Singapore.

### 4.6 Diabetes

**Figure 6:**
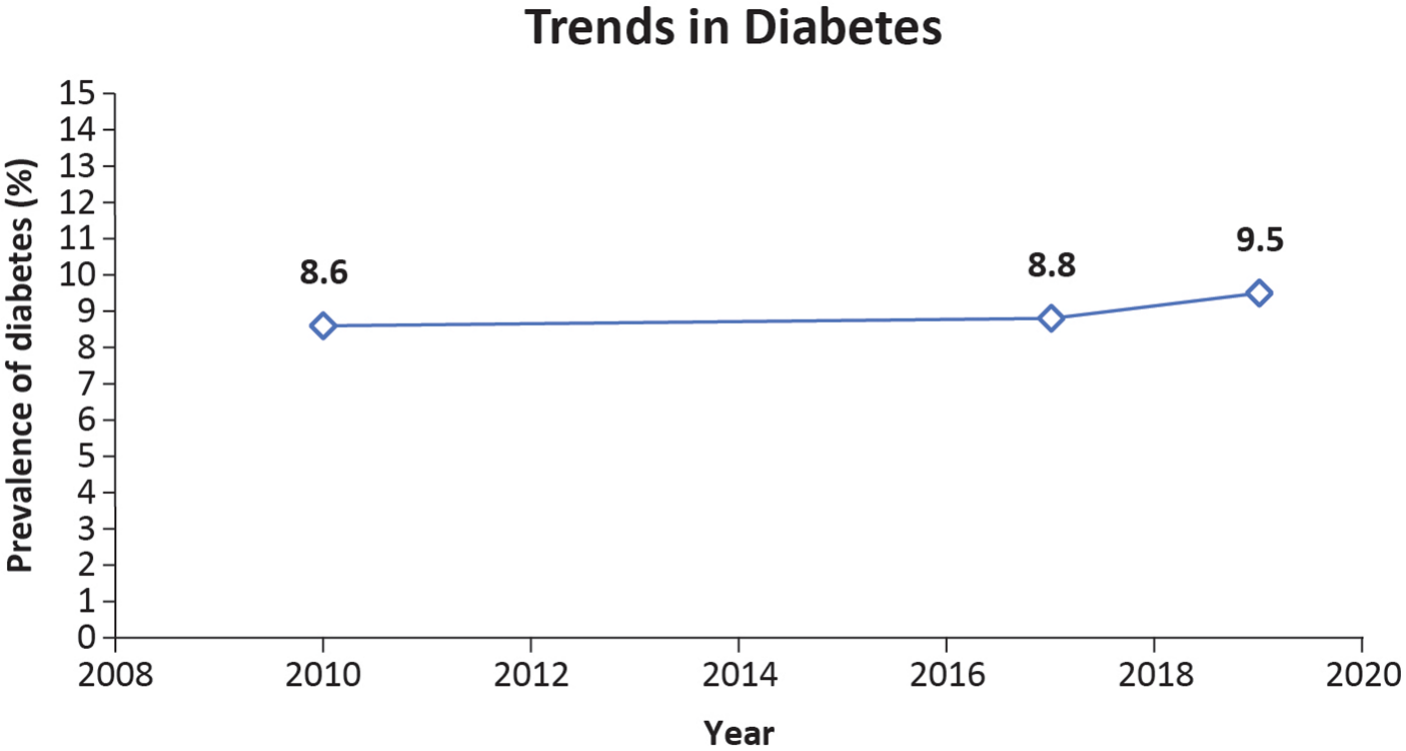
Trends in the prevalence of diabetes in Singaporeans aged 18–74 years (21).

Type II diabetes mellitus is associated with accelerated atherosclerosis, which is one of the causes of morbidity and mortality (26). Singapore's National Population Health Survey 2020 reported an increase in the prevalence of diabetes (Figure 6) (21). This percentage is projected to increase further to 13.3% by 2030 and 14.3% by 2045 (27). The age-adjusted prevalence of diabetes (20–79 years) in 2021 was 11.6%. Undiagnosed diabetes accounted for 50.4% of the population in 2021 (27). Ethnic minorities are more likely to have diabetes mellitus in Singapore than Chinese minorities. It has been reported that 16.7% of Malays and 17.7% of Indians have diabetes compared to 9.7% of Chinese individuals (5). Malays were more likely to have undiagnosed diabetes, whereas Indians showed a higher level of insulin resistance (14). Individuals of Chinese ethnicity are at a particular risk of developing diabetes mellitus and metabolic diseases due to rising obesity, whereas obesity-independent pathways may play a significant role in Asian Indians (14).

Diabetes mellitus has also been found to be a risk factor for the development of vascular disease and is associated with adverse outcomes in acute MI and stroke, according to Yeo et al. As of 2010, 43.8% of AMI patients and 43.1% of stroke patients in Singapore had diabetes mellitus, which is significantly higher than the national prevalence (11.3%) (22).

### 4.7 Hypertension

**Figure 7:**
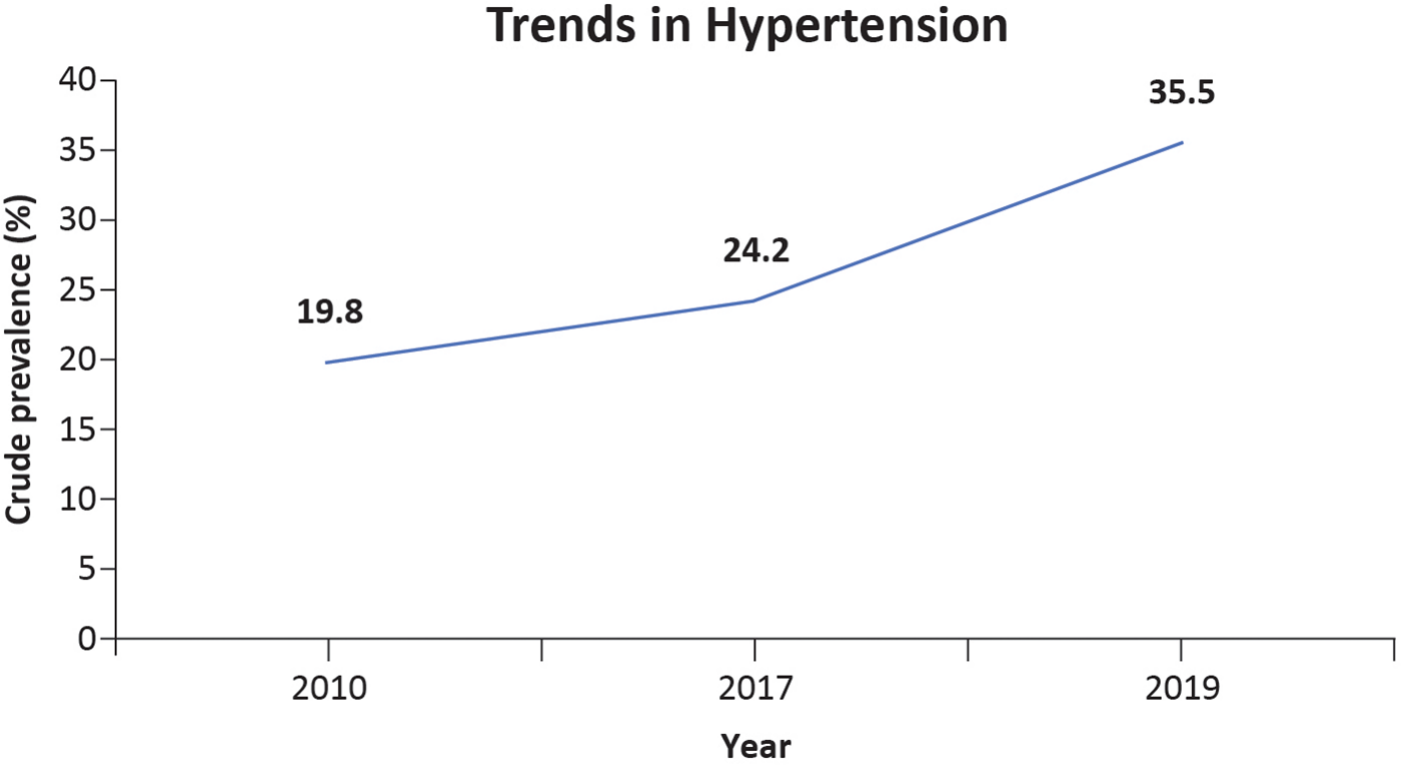
Trends in the prevalence of hypertension in Singaporeans aged 18–74 years (21).

Hypertension is a common modifiable risk factor for CVD is hypertension (28). The age-standardized prevalence of hypertension in Singapore has increased from 21.9% in 2017 to 31.7% in 2020. People aged 40–59 years, particularly Chinese and Malay people, experienced the greatest increase in hypertension. An estimated third of adults with an early diagnosis of hypertension (35.7%) had good control over their blood pressure, whereas the remaining 64.3% of the population showed less effective control (21). A greater proportion of Malay and Chinese people had hypertension compared to 29.5% of Indians (21). Additionally, Malays were more likely to be unaware of hypertension and even when patients were adequately aware of their hypertensive state, blood pressure control remained poor (14). The trends in the prevalence of hypertension in Singaporeans are shown in Figure 7.

Approximately 62% of strokes and 49% of cardiovascular deaths are caused by high blood pressure (16). The use of antihypertensive therapies can reduce the relative and absolute risks of CVD-associated mortality and its complications (28). Kang et al. found that nearly one in two patients had poor adherence to antihypertensive medications, which indirectly contributed to stroke and IHD deaths (29).

## 5 Change in trends of ASCVD in terms of the epidemiological transition

A clear understanding of the epidemiological environment of the country is essential for reducing ASCVD. With rapid development since its independence, Singapore has moved from being a low-income to a high-income country. The gross domestic product (GDP) growth has been among the highest, with an average 7.7% increase each year (30). Due to economic development, the country saw an improvement in LE. The average LE increased from 62 years in 1957 to 84 years in 2022 (6, 31). During this period, disease patterns also changed, indicating an epidemiological transition from communicable to noncommunicable diseases. The epidemiological transition model outlines the different stages through which populations pass as they undergo social and economic development (32). The low- and middle-income countries in Asia are grappling with infectious diseases, while also beginning to address the rising incidence of chronic conditions (33). Countries such as Japan, South Korea, and Singapore have moved to stage 3 of the epidemiological transition model with a shift towards chronic noncommunicable diseases with challenges related to aging populations and lifestyle-related health issues (34). Age-adjusted mortality rates for CHD rose from 27 per 100,000 individuals in 1957 to 108 per 100,000 individuals in 2012 (6, 35). This is likely due to improved LE, an aging population, and societal changes such as improvements in housing, sanitation, water, vaccinations, nutrition, and equitable access to healthcare. Dietary habits are changing, sedentary lifestyles are increasing, and exercise is lacking (6, 14). Furthermore, different ethnic groups displayed different disease rates within the same population. AMI mortality rates vary among different ethnic groups in Singapore, with Chinese (22.1) having lower values than Malays (51.7) and Indians (33.6) (35).

While ethnic groups may show variations in CHD due to varying environmental factors (diet, smoking, and physical activity), there may be differences that cannot be explained by these traditional risk factors, such as greater susceptibility to CHD among Indians (36). High abdominal obesity and insulin resistance may explain this variation. Genetic factors may also play a role in the interpopulation differences. Malays have a higher case-fatality rate than Indians. There is no clear explanation for this, especially in Singapore, where healthcare is readily available for the entire population (6).

## 6 The burden of ASCVD: disability, work absenteeism/presenteeism, and early retirement

The impact of CVD on individuals, their households, and public finances is crucial to understanding the impact of the disease (1, 37). To understand the burden of disease in the country, it is critical to understand the direct and indirect costs of hospitalization, long-term management via medications, functional impairment, and their impact on productivity. In 2020, Singapore's expenditure on CVD costs amounted to 8.1 billion USD (equivalent to 11.5 billion SGD) (37). The costs of hypertension, hypercholesterolemia, obesity, and smoking in Singapore are estimated at 4.9 billion USD (38). Peripheral vascular diseases also contribute to a substantial economic burden in the Asia-Pacific region (39). The direct costs of CVD are hospitalization, drugs, rehabilitation, and outpatient care, whereas indirect costs are productivity loss, informal care, and early retirement (38).

It is now recognized that an elevated LDL-C level is a major modifiable risk factor for ASCVD (8). Various studies have confirmed that lowering LDL levels is associated with better cardiovascular outcomes (40). The prevalence of dyslipidemia among adults in Singapore is approximately 48% (21). Since dyslipidemia plays a pivotal role in the development of atherosclerosis and its associated clinical events, its management has become a high priority in cardiovascular prevention.

### 6.1 Direct costs

Singapore's hospitalization costs are heavily influenced by whether the patient receives care in the public or private sector. In March 2022, IHD costs an average of 2,944 SGD in public subsidized wards, 11,618 SGD in public unsubsidized wards, and 24,149 SGD in private hospitals (41). A systematic review found that routine management of CVD costs 563 dollars per person per year (or 755.76 SGD per person per year). The price increases further if complications occur (42). The cost of generic medication for hyperlipidemia ranged from 2.7 SGD to 3.05 SGD per pill. Non-generic or branded medications, on average cost 1.4 times more than generic medication. Non-generic statins cost between 3.93 SGD and 4.44 SGD per pill (41). To minimize the economic burden of living with CVD, policymakers must focus on prevention, early diagnosis, and prompt treatment to mitigate the high direct costs associated with managing CVD (42).

### 6.2 Indirect costs

Strokes and IHD can leave survivors with persistent disabilities. Stroke is a common cause of disability among Singaporeans aged ≥ 65 years and is associated with long-term dependence on healthcare services. Older patients with occasional care requirements were four times more common, and those with severe care needs were ten times more common among stroke patients than among those without stroke. Owing to inconvenience, cost, or lack of interest, many stroke patients do not undergo post-stroke therapy. It is estimated that approximately 95% of Singapore's older adults live with their families, and if they are disabled, they become more dependent on their families. This emphasizes the need to redesign existing support services for stroke survivors (43).

### 6.3 Work disabilities

IHD often develops at a productive age, resulting in work disabilities and absences. Work disabilities were more prevalent among individuals with comorbid conditions such as diabetes, musculoskeletal disorders, and mental disorders. Individuals with IHD may also experience depression, which can further increase their work disability (44). Improved treatment, declining mortality, and the pressure of extended working careers may lead to an increase in CVD among working-age individuals. Stroke can lead to permanent disability (45). Understanding trends in work disability can help policymakers plan interventions to assist people in returning to work (44).

### 6.4 Absenteeism/presenteeism

Work disability, absenteeism, early retirement, and tax loss have resulted in indirect costs of 4.6 billion USD Singapore (38). According to a 2017 report, early retirement due to poor health leads to loss of productivity, costing 2.2% of the GDP; absenteeism and presenteeism cost 3.2% of the GDP; and presenteeism, absenteeism, and early retirement cost 5.4% of the GDP. The estimates for 2030 are as follows: early retirement due to poor health leads to loss of productivity, accounting for 2.3% of the GDP; absenteeism and presenteeism cost 3.4% of GDP; and presenteeism, absenteeism, and early retirement cost 5.7% of the GDP. As a result of early retirement due to CVD, the government has to probably reallocate funds from other budgets to healthcare to cover the rising costs (46). The issues of absenteeism and presenteeism in an aging workforce due to ill health can have implications for decisions regarding the revision of the retirement age. Such issues pose challenges in terms of the costs for organizations to maintain the well-being and performance of employees across their working lives (47). Therefore, the key to reducing the CVD burden is to focus on primary prevention among high-risk groups and on secondary prevention among survivors.

## 7 Risk trend analysis and recommendations

ASCVD continues to be a major contributor to death and disability in Singapore, with economic consequences for affected individuals and the country at large. However, most risk factors for ASCVD are preventable. By understanding the burden of ASCVD, trends in risk factor control, preventive care, disparities, and current unmet management needs of ASCVD in Singapore, this landscape aims to address recent challenges in the control of ASCVD.

A study by Lopez AD and Adair Tim on the recent trends of age-standardized CVD mortality in high-income countries and their relationship to the trends in associated risk factors suggested three important points. First, after an initial rapid decline in ASMR, the decline in CVD-related ASMR gradually slowed down, which is a general phenomenon exhibited in countries with diverse CVD epidemiological environments, such as Germany, Denmark, Australia, Italy, Spain, the USA, and the UK. Second, the trends were similar in males and females. Third, the momentum in the change of trends is consistent, suggesting that these countries are likely to follow the trend and present a possible increase in mortality, as seen in the USA (7). Table 1 compares the trends in ASMR between the USA States and Singapore. These findings are particularly important, as they suggest that certain risk factors contribute to the initial rapid change, while others need to be revisited to facilitate continued deceleration in CVD-related ASMR.

**Table 1:** Comparison of ASMR in Singapore and the USA (7).

Country	Years	Average annual change to 2010 (%) males	Average annual change since 2010 (%) males	Latest annual change (%) males
USA	2000–2016	−3.5	−0.8	−0.1
Singapore	2000–2016	−4.2	−3.4	−2.4

A study on the prevalence of major CVD risk factors in Singapore indicates that there is still much to be done. Tobacco use declined overall, which may have contributed to the initial decrease in the ASMR values. However, there has been a gradual increase in the number of female smokers. To reverse this worrying trend, new public health measures are required. Despite various health programs, the incidence of hypertension, diabetes, and obesity is on the rise. This could be attributed, in part, to the shift from traditional diets to westernized, high-calorie, high-fat, and high-sodium diets. Physical activity decreases concomitantly, resulting in obesity and metabolic syndromes.

Singapore is also in the third stage of the epidemiological transition, which corresponds to improved LE with worsened lifestyle changes such as high-fat diets, smoking, and sedentary behaviors (48).

## 8 Individual and population-based strategies for prevention and management of ASCVD: opportunities and challenges

Individual strategies work to identify at-risk individuals, especially due to lifestyle habits, and counsel them for change. These have been less successful. Population strategies require multi-sectoral efforts to change the population's behavior or environment to reduce exposure to underlying risk factors. These two approaches can be used in tandem for mutual support.

### 8.1 Individual approaches

ASCVD can be managed successfully using various guidelines. Patients with a low cardiovascular disease risk may benefit from lifestyle modifications without medication (49). Physical activity and a diet containing whole grains, fresh fruits and vegetables, and low-fat dairy are beneficial for controlling obesity (50). Other approaches included quitting smoking, reducing stress, and focusing on nutrition. In the PREMIER trial, Maruthur et al. found that lifestyle modifications could reduce CHD risk by 12%–14%, reiterating the importance of individual patient care (51). Guidelines also follow a treat-to-target approach, statin intensity-centric approach, or a combination of these approaches (49). As of 2017, 47% of Singaporeans used low-potency statins, usually prescribed by general practitioners (49).

Another study by Poh et al. found that a proportion of patients with high cardiovascular risk had concurrent severe hyperlipidemia that was poorly managed. In Singapore, low-intensity lipid-lowering therapy is widely used among patients with CHD and acute coronary syndrome. The average atorvastatin-equivalent dosage prescribed was often low, and combination therapies were rarely used. Healthcare practitioners in Singapore are increasingly prescribing simvastatin due to its lower cost and greater availability. Healthcare practitioners in Singapore are increasingly prescribing simvastatin due to its lower cost and greater availability. The lack of awareness of the 2013 American College of Cardiology/American Heart Association (ACC/AHA) guidelines, the belief that Asian populations are less tolerant to high-dose statins, and the fear of dose-dependent side effects are also contributing factors (52).

Gan suggested that preventing cardiovascular events in adults with an LDL-C of ≥190 mg/dl may be achieved with high-intensity statins. In adults with clinically established ASCVD, age becomes a crucial factor. For patients aged 75 years and above, high-intensity statins are recommended, while for those below 75 years, moderate-intensity statins are recommended. For patients aged 75 years and above, high-intensity statins are recommended, while for those below 75 years, moderate-intensity statins are recommended. For patients with type 1 and 2 diabetes mellitus, aged between 40 and 75 years, having an LDL-C of 70–190 mg/dl and a 10-year ASCVD risk of <7.5%, a moderate-intensity statin is recommended. Conversely, for patients with a 10-year ASCVD risk of >7.5%, a high-intensity statin is advised. According to the authors, it is preferable to consider high-intensity statin therapy, moderate-intensity statin therapy, and low-intensity statin therapy, rather than focusing solely on LDL-C goals (35).

There is a paucity of trials evaluating treatment targets or ongoing treatment monitoring on the commencement of treatment. This limitation may stem from the high costs associated with conducting such trials. Generally, all guidelines recommend commencing treatment with lipid-lowering drugs based on randomized controlled trial data, though some evidence states that statins are not consistently prescribed within the secondary prevention population depending on the cardiovascular events experienced. The recommendation is to initiate therapy with a high dose and subsequently down-titrate it as needed (53). Nevertheless, certain high-risk patients may exhibit elevated LDL-C despite statin therapy. To cater to the needs of these patients, treatment modalities, such as ezetimibe and anti-proprotein convertase subtilisin/kexin type 9 (PCSK9) monoclonal antibodies (mAbs), are also existent. The European Society of Cardiology recommends the use of ezetimibe when patients fail to achieve specific LDL targets (54). Small interfering ribonucleic acid (siRNA)-based drug targeting PCSK9 mRNA and anti-PCSK9 mAbs are additional therapeutic agents. PCSK9 is a hepatic protease that binds to LDL receptors, facilitating their internalization into lysosomes and subsequent degradation. Two such monoclonal antibodies (mAbs), namely alirocumab and evolocumab, received approval from the Food and Drug Administration in 2015 (55). A systematic review of lipid-lowering therapies and subsequent network meta-analysis concluded that combining anti-PCSK9 mAB with medium or high-intensity statin therapy demonstrated substantial reductions in LDL-C levels. Among the anti-PCSK9 mAbs assessed, evolocumab exhibited a more pronounced reduction in LDL-C compared to alirocumab. The studies included in the analysis did not observe any evidence of a loss in efficiency or increased rates of adverse events, even upon long-term follow-up (56). Inclisiran is a long-acting siRNA targeting PCSK9 mRNA that is administered by the subcutaneous route. It reduces both intra- and extrahepatic PCSK9 levels, resulting in a significant and sustained reduction of LDL-C levels. Its advantage also lies in its favorable administration regimen, consisting of an initial dose followed by a second dose at 3 months, followed by biannual subcutaneous dosing thereafter (57). The use of inclisiran has recently been approved in Singapore for the management of hypercholesterolemia (58). However, these therapies are yet to find a place in the Clinical Practice Guidelines of the Ministry of Health, Singapore, leaving an unmet need open for new therapies, capable of achieving LDL-C reduction beyond that accomplished by statins (25).

In Singapore, poor management of hypertension was mainly observed among elderly individuals from low socio-economic strata. Barriers to effective management could be inaccessibility to home monitoring, lack of knowledge, ineffective communication with healthcare professionals, and poor social support. However, financing, seems to be the main barrier to treatment adherence. In high-income countries, the burden of disease is concentrated at the lower rungs of the social ladder. Addressing this issue requires multi-sectoral policy decisions rooted in the principles of health equity (59).

There is evidence supporting the use of fixed-dose therapy during the initial treatment of CVD, along with regular monitoring of patients, which enhances medication adherence and improves their health outcomes (53, 60). It has been suggested that advances in electronic health records (EHRs) could facilitate evidence-based practice, particularly given the high costs associated with trial monitoring (53). This technology could be an integral part of delivering consistent, accurate, and individualized recommendations to patients with ASCVD. According to guidelines and patient-specific data in the EHR, Mayo Expert Advisor provides CVD risk scores and treatment recommendations. This tool streamlines the calculation of ASCVD risk scores and facilitates cholesterol treatment by making the process more efficient and precise (61).

Using telemedicine for medication adherence depends on the patient's persona. Through the use of mobile health (mHealth) services, Haldane et al. explored various user behaviors and preferences. Personas were developed to categorize diverse user experiences, aiming to facilitate the design and creation of mHealth interventions for ASCVD medication adherence in patients of Singapore with ASCVD. Five distinct types of personas were identified: (a) the Quiet Analog, (b) the Busy Grandparent, (c) the Socializer, (d) the Newly Diagnosed, and (e) the Hard-to–Reach. The Quiet Analog persona describes individuals who shy away from technology and do not actively seek out health information but may benefit from the introduction of mHealth by healthcare providers. The Busy Grandparent persona represents individuals who regularly use their phones but may not trust mHealth information. Nevertheless, if their polyclinics suggest mHealth to them, they may respond positively to it. A Socializer persona is more inclined to use mHealth if it is tailored to their needs, given their frequent use of smartphones. Newly diagnosed patients with chronic conditions require maximum support, and mHealth aligns well with their needs. However, individuals categorized as Hard-to–Reach may face challenges with mHealth adoption due to social and technological disconnection. This group may struggle with literacy, making the utilization of mHealth difficult for them. Mobile short message services may be of benefit to this demographic. Singapore has a 91% mobile ownership rate, which provides a conducive environment to mHealth interventions (62). However, acceptance of mobile technology varied among participants. Participants showed limited interest in using mHealth to aid in medication adherence. Some felt they did not need technology to remind them, and others were unsure that the other party would not take advantage of them (63).

Increasing healthcare spending alone will not suffice to address this multifaceted issue. To achieve a value-based care, a new model must emerge that moves away from episodic, fee-for–service care. It requires a holistic approach to patient treatment, emphasizing healthcare integration, strengthening primary healthcare services, and collaborating with healthcare providers in the community (64).

### 8.2 Population strategies

One of the challenges in CVD management in Singapore is the lack of awareness about the factors contributing to CVD. Notably, lack of awareness regarding hypertension (43.7%) appeared to be the most common, followed by poor awareness regarding hypercholesterolemia (40.9%) and diabetes (30.7%). Among the diverse ethnicities residing in Singapore, Malays appeared to have the lowest awareness of the risk factors for CVD. Low awareness was associated with an increased prevalence of hypertension and hypercholesterolemia. Despite lacking awareness about their condition, elderly Singaporeans who relied on caregivers were found to receive suitable medication. Simple language and easy-to–understand public health initiatives could assist in raising awareness and empowering the elderly to engage in self-care (65).

The Health Promotion Board initiated the Healthy Dining Program in Singapore to encourage healthy food and beverage (F&B) consumption. This initiative involved collaboration with private sector F&B companies to provide healthier meal options. To incentivize F&B outlets to participate in the program, the Singapore government offered grants of 3,000 SGD for marketing and publicity costs. The F&B outlets had to offer at least one healthy F&B option to qualify for the program. The program saw a remarkable 300% increase in sale of healthy meals from 2014 to 2017; furthermore, since its inception, more than 2,000 partners have joined the program (64).

Psychological aspects, such as stress and depression, along with socio-economic issues, are recognized as risk factors for CVD. However, risk factors such as poverty and low education may not be recognized. Currently, the focus in CVD management predominantly revolves around prolonging survival after CVD. However, there should be an emphasis on improving the quality of life (QoL) by reducing symptoms, optimizing daily activities and enhancing well-being. Cardiac rehabilitation programs with lifestyle modifications, psychological interventions, and counselling could limit the physiological and psychological effects of CVD and enhance QoL (66). Additionally, continuous public health efforts are essential to address treatment gaps among patients with diabetes and individuals with poorly controlled vascular risks in Singapore (23).

ASCVD could have its beginnings in childhood and slowly progress throughout life. This progression can be halted, and maybe even reversed to some extent (67). There is a pressing need for more research on risk stratification to identify high-risk features, enabling the initiation of more targeted and personalized preventative interventions (68).

Early childhood and adolescent risk factors have the potential to predict the development of clinical atherosclerotic disease by midlife. Thus, implementing “primordial prevention” strategies, beginning right from maternal health and early childhood through adolescence, is crucial for reducing these risk factors.

## 9 Future directions

ASCVD is the most common type of CVD in Asia, with many countries, including Singapore experiencing a rise in its prevalence. LE and lifestyle changes increase the risk of ASCVD. Disability and absenteeism can have significant impacts on individuals affected by ASCVD. The recommended treatment for ASCVD differs with different guidelines, but statins are usually prescribed for managing ASCVD in susceptible patients. However, further studies are required to demystify the efficacy of statins among Asian population. Additionally, high-risk patients may benefit from statins alone or in combination with PCSK9-targeting agents, such as mAbs and siRNA. Besides the controversy surrounding the differing guidelines, another challenge in ASCVD management is patients' non-adherence to the medication regimens. mHealth technology is gaining significant momentum and has proven beneficial in ensuring treatment adherence among CVD patients. The Singapore Government has played a significant role in the prevention and management of ASCVD through schemes such as the “Healthier Dining Program” and the “National Steps Challenge”.

## 10 Conclusion

As the burden of CVD continues to rise in Singapore, there is an increased need to prioritize on healthcare services that are specific to its needs. The impact of common as well as geographic and ethnicity-specific influences on the health of its people suggests the need for scientific investigations into population health. Such investigations should serve as the basis of decision-making associated with public health policies.
